# Myeloid-Specific Deletion of Mcl-1 Yields Severely Neutropenic Mice That Survive and Breed in Homozygous Form

**DOI:** 10.4049/jimmunol.1701803

**Published:** 2018-11-21

**Authors:** Janka Zsófia Csepregi, Anita Orosz, Erik Zajta, Orsolya Kása, Tamás Németh, Edina Simon, Szabina Fodor, Katalin Csonka, Balázs L. Barátki, Dorottya Kövesdi, You-Wen He, Attila Gácser, Attila Mócsai

**Affiliations:** * Department of Physiology, Semmelweis University School of Medicine, 1094 Budapest, Hungary;; † MTA-SE “Lendület” Inflammation Physiology Research Group of the Hungarian Academy of Sciences and Semmelweis University, 1094 Budapest, Hungary;; ‡ Department of Microbiology, University of Szeged, 6726 Szeged, Hungary;; § Department of Computer Science, Corvinus University of Budapest, 1093 Budapest, Hungary;; ¶ Department of Immunology, Eötvös Loránd University, 1117 Budapest, Hungary;; ‖ Office of Supported Research Groups of the Hungarian Academy of Sciences, 1051 Budapest, Hungary; and; # Department of Immunology, Duke University Medical Center, Durham, NC 27710

## Abstract

Mouse strains with specific deficiency of given hematopoietic lineages provide invaluable tools for understanding blood cell function in health and disease. Whereas neutrophils are dominant leukocytes in humans and mice, there are no widely useful genetic models of neutrophil deficiency in mice. In this study, we show that myeloid-specific deletion of the Mcl-1 antiapoptotic protein in *Lyz2*^Cre/Cre^*Mcl1*^flox/flox^ (*Mcl1*^ΔMyelo^) mice leads to dramatic reduction of circulating and tissue neutrophil counts without affecting circulating lymphocyte, monocyte, or eosinophil numbers. Surprisingly, *Mcl1*^ΔMyelo^ mice appeared normally, and their survival was mostly normal both under specific pathogen-free and conventional housing conditions. *Mcl1*^ΔMyelo^ mice were also able to breed in homozygous form, making them highly useful for in vivo experimental studies. The functional relevance of neutropenia was confirmed by the complete protection of *Mcl1*^ΔMyelo^ mice from arthritis development in the K/B×N serum-transfer model and from skin inflammation in an autoantibody-induced mouse model of epidermolysis bullosa acquisita. *Mcl1*^ΔMyelo^ mice were also highly susceptible to systemic *Staphylococcus aureus* or *Candida albicans* infection, due to defective clearance of the invading pathogens. Although neutrophil-specific deletion of Mcl-1 in *MRP8*-Cre*Mcl1*^flox/flox^ (*Mcl1*^ΔPMN^) mice also led to severe neutropenia, those mice showed an overt wasting phenotype and strongly reduced survival and breeding, limiting their use as an experimental model of neutrophil deficiency. Taken together, our results with the *Mcl1*^ΔMyelo^ mice indicate that severe neutropenia does not abrogate the viability and fertility of mice, and they provide a useful genetic mouse model for the analysis of the role of neutrophils in health and disease.

## Introduction

Genetically manipulated mice lacking a certain hematopoietic lineage (1–11) have strongly contributed to our understanding of immune and inflammatory processes in health and disease. The best example is the deficiency of the recombination activating genes *Rag1* or *Rag2*, which lack B and T lymphocytes and, therefore, are widely used to test the role of the adaptive immune response in in vivo biological processes (1). Additional mutations result in the deficiency of B cells (2), T cell subtypes (3, 4), NK-cells (4), eosinophils (7), basophils (8), mast cells (9, 10), or certain macrophage lineages (11), allowing the analysis of those lineages in the immune and inflammatory process. The usefulness of such models is determined by the extent and selectivity of the deficiency of the given lineage as well as general characteristics, such as the survival and breeding of the mutant mice.

Neutrophils are the most abundant circulating leukocytes in humans and a predominant leukocyte population in experimental mice. Neutrophils are critically involved in the innate immune response, but they also contribute to tissue damage upon inappropriate activation of the cells (12–15). There are a number of mouse strains that show reduced numbers of neutrophils due to mutations in the genes encoding the Gfi1 transcription factor (16–18), G-CSF (19), G-CSF receptor (20), or the Foxo3A transcription factor (21). Unfortunately, all those models have substantial limitations, such as poor specificity (16, 17), partial neutrophil deficiency, (18–21) or limited survival of the affected animals (16, 17, 19, 20). In addition, although it is widely believed that severe neutropenia is inconsistent with life, this has never been appropriately tested in experimental mice.

Mcl-1 (myeloid cell leukemia 1) is an antiapoptotic member of the Bcl-2 family protein present in various tissues (22, 23). We have previously shown that Mcl-1 is required for the survival of neutrophils (24), likely because these short-lived cells lack other antiapoptotic Bcl-2 family members able to control the intrinsic proapoptotic program of neutrophils (25). In contrast, the survival of other myeloid cells, such as macrophages, does not rely on Mcl-1 expression (24), likely because those cells also express antiapoptotic proteins other than Mcl-1.

Given the critical role of Mcl-1 in neutrophil but not macrophage survival, we hypothesized that myeloid-specific deletion of Mcl-1 would lead to selective loss of neutrophils but not of monocytes/macrophages or nonmyeloid lineages. Indeed, Cre/lox–mediated myeloid-specific deletion of Mcl-1 led to very severe neutropenia without affecting other hematopoietic lineages. Surprisingly, the survival and fertility of these mice was mostly normal, indicating that mice are able to survive with very low circulating neutrophil numbers. This mouse strain may be suitable for the analysis of the role of neutrophils in various in vivo biological processes in health and disease.

## Materials and Methods

### Animals

Mice carrying the *Mcl1*^tm1Ywh^ (*Mcl1*^flox^) floxed allele of the Mcl-1–encoding gene (24) were crossed to mice carrying the *Lyz2*^tm1(cre)Ifo^ (*Lyz2*^Cre^; also known as LysM-Cre) knock-in strain expressing the Cre recombinase in the entire myeloid compartment (26) to generate *Lyz2*^Cre/Cre^*Mcl1*^flox/flox^ mutants (referred to as *Mcl1*^ΔMyelo^ mice). The mutations were mostly maintained by breeding *Mcl1*^ΔMyelo^ with *Lyz2*^Cre/Cre^*Mcl1*^flox/+^ mice, yielding *Mcl1*^ΔMyelo^ homozygous animals and *Lyz2*^Cre/Cre^*Mcl1*^flox/+^ littermate controls. Several other breeding strategies (including breeding in the *Mcl1*^ΔMyelo^ homozygous form) were also used (see *Results*). To generate a more neutrophil-specific Mcl-1 deletion, *Mcl1*^flox/flox^ mice were crossed to *MRP8*-Cre transgenic animals (27) to generate *MRP8*-Cre*Mcl1*^flox/flox^ (referred to as *Mcl1*^ΔPMN^) mice. G-CSF receptor–deficient (20) (*Csf3r*^tm1Link/tm1Link^; *Csf3r*^−/−^) mice were purchased from The Jackson Laboratory. The genotype of all mice was tested by allele-specific PCR. All mice were on the C57BL/6 genetic background. Control C57BL/6 animals were obtained from our breeding colony.

Mice were kept in individually sterile ventilated cages (Tecniplast), either in a specific pathogen-free facility or an adjacent conventional facility. The conventional facility has historically been infected with murine hepatitis virus, Theiler murine encephalomyelitis virus, and murine norovirus as well as with *Helicobacter*, *Entamoeba*, *Hexamastix*, *Syphacia obvelata*, and *Mycoptes musculinus* species. All experiments were approved by the Animal Experimentation Review Board of Semmelweis University or the University of Szeged. Mice of both genders at 2–6 mo of age were used for the experiments.

Bone marrow chimeras were generated by i.v. injection of unfractionated bone marrow cells into B6.SJL-*Ptprc*^a^ recipients carrying the CD45.1 allele on the C57BL/6 background lethally irradiated by 11.5 Gy from a [^137^Cs] source using a Gamma-Service Medical (Leipzig, Germany) D1 irradiator. Four weeks after transplantation, peripheral blood samples were stained for Ly6G and CD45.2 and analyzed by flow cytometry. Bone marrow chimeras were used 4–10 wk after the transplantation.

### Abs

The following Abs (all from BD Biosciences, except 7/4 from Abcam and IgM from Jackson Immonoresearch) were used for flow cytometry: CD3 (17A2), CD11b (M1/70), CD45R/B220 (RA3-6B2), CD45.2 (104), Ly6C (AL-21), Ly6G (1A8), Siglec-F (E50-2440), Gr1 (RB6-8C5), 7/4 (ab53453), c-Kit (2B8), B220 (RA3-6B2), IgM (polyclonal, catalog no. 115-606-020), IgD (11-26c.2a), CD21 (7g6), and CD23 (B3B4).

### Cell preparation, flow cytometry, and cytospin

Blood samples were obtained from tail vein incisions, washed, stained, and then resuspended in BD Biosciences FACS lysing solution. Bone marrow and spleen cell samples were obtained by flushing the bone marrow or crushing the spleen through a 70-μm cell strainer, followed by RBC lysis with eBioscience RBC Lysis Buffer, staining, and resuspension in PBS containing 5% FBS. Samples were kept at 4°C during the entire procedure. Specified volumes were used throughout, allowing a precise determination of absolute cell counts.

Flow cytometry was performed using a BD Biosciences FACSCalibur and analyzed by FCS Express 6 (De Novo Software). The different leukocyte populations were identified within their typical forward and side scatter gates as follows: neutrophils as CD11b^+^Ly6G^+^Siglec-F**^−^**, monocytes as CD11b^+^Ly6G^–^Siglec-F**^−^**, eosinophils as Ly6G**^−^**Siglec-F^+^, T cells as CD3^+^, and B cells as B220^+^ cells. Blood monocyte subpopulations were differentiated by Ly6C staining.

For cytospin assays, bone marrow cells were obtained by flushing the bone marrow, followed by RBC lysis with eBioscience RBC Lysis Buffer. Cell counts were adjusted and cytospined onto SuperFrost slides (Thermo Fisher Scientific) for 5 min at room temperature using Shandon Cytospin 3 Cytocentrifuge cytospin equipment. After drying, slides were stained with the May–Grünwald method and analyzed by a Leica DMI6000B inverted microscope.

### In vitro culture and PCR analysis of macrophages

Bone marrow cells were obtained by flushing the bone marrow. Cells were washed and resuspended in α-MEM supplemented with 10% FBS, 1% penicillin/streptomycin, 10 mM HEPES (pH 7.4), 1% l-glutamine, and 10 ng/ml recombinant murine M-CSF. Cells were plated on tissue culture–treated plates and cultured for 3 d in a humidified CO_2_ incubator. Cells in suspension were then collected, centrifuged, and resuspended in the above-mentioned medium containing 40 ng/ml recombinant murine M-CSF. Four days later, adherent cells were collected and prepared for flow cytometry using the F4/80 marker or isolation of genomic DNA. For *Mcl1* genomic PCR analysis, the 5′-GGT TCC CTG TCT CCT TAC TTA CTG TAF-3′ forward primer was used along with the 5′-TCG AGA AAA AGA TTT AAC ATC GCC-3′ reverse primer (*Mcl1*^Δ^ allele; ∼600-bp product length) or the 5′-CTC CTA ACC ACT GTT CCT GAC ATC C-3′ reverse primer (*Mcl1*^WT^ or *Mcl1*^flox^ allele; ∼260- and 380-bp product length, respectively). For *Itgb2* (CD18) PCR analysis, the 5′-GCC CAC ACT CAC TGC TGC TTG-3′ forward primer was used along with the 5′-CCC GGC AAC TGC TGA CTT TGT-3′ reverse primer (*Itgb2*^WT^ allele; ∼480-bp product length).

### Thioglycolate-induced peritonitis

Peritonitis was induced by i.p. injection of 1 ml 3% thioglycolate (Liofilchem) or PBS. After 4 h, mice were euthanized, and the peritoneum was flushed by 5 ml ice-cold PBS containing 5% FBS. The lavage samples were washed, resuspended in PBS containing 5% FBS, and maintained at 4°C until staining for flow cytometry.

### Survival and fertility

An online database (specific pathogen-free facility) and hand-written records (conventional facility) were used for the analysis of the survival, fertility, and breeding behavior of our mice. Data were analyzed using a custom-made software. Body weight of a smaller cohort was measured once weekly from the age of 2 wk.

### K/B×N serum transfer arthritis

Serum from KRN transgene-positive (arthritic) K/B×N and transgene-negative (nonarthritic) B×N mice was obtained as described previously (28, 29). Arthritis was induced by i.p. injection of 300 μl K/B×N (arthritic) or B×N (control) serum, followed by daily scoring of clinical signs of arthritis and measurement of ankle thickness for 2 wk as described previously (29–32).

### Autoantibody-induced skin-blistering model

The murine model of human epidermolysis bullosa acquisita was triggered by systemic administration of rabbit polyclonal Abs against type VII collagen (CVII) as described previously (31–34). Twelve milligrams of pathogenic IgG in PBS per mouse or PBS alone was injected s.c. under isoflurane anesthesia every second day between 0 and 8 d (60 mg total IgG per mouse). The disease onset and progression were followed by clinical assessment every second day as described previously (31, 32, 34).

### In vivo infection models

*Staphylococcus aureus* strain ATCC25923 and *Candida albicans* strain SC5314 originated from the Szeged Microbial Collection (World Federation of Culture Collections no. 987).

*S. aureus* was maintained on brain–heart infusion (BHI) agar and grown overnight at 37°C in liquid BHI medium prior to experiments. Mice were infected i.p. with 2 × 10^7^ or 1 × 10^7^ *S. aureus* bacteria in 100 μl PBS per mouse for survival assays and bacterial burden assessment, respectively.

*C. albicans* was maintained on yeast extract/peptone/dextrose (YPD) agar and grown overnight at 30°C in liquid YPD medium prior to experiments. Mice were infected i.v. through the tail vein with 1 × 10^5^ yeast cells in 100 μl PBS per mouse.

Bacterial and fungal burdens were determined by a conventional CFU counting method 12 h post infection. Kidneys, spleens, livers, and brains were collected, weighed, and homogenized in sterile PBS. Blood was also collected from the retro-orbital venous plexus. Peritoneal lavage was collected by washing the peritoneum with 5 ml sterile PBS. Samples were plated in serial dilutions on BHI or YPD agar plates and incubated for 1 d at 37°C or for 2 d at 30°C, respectively, followed by CFU counting.

### Presentation of data and statistical analysis

Experiments were performed the indicated number of times. Bar graphs and kinetic curves show mean and SEM of all mice or samples from the indicated number of independent experiments. Tissue cell numbers were calculated for the entire spleen, the entire peritoneum, or the bone marrow of both femurs and both humeri combined. Statistical analysis was performed with StatSoft Statistica software. The analysis of blood, bone marrow, and splenic leukocyte populations and bacterial or fungal CFU counts was performed by Student *t* test. Peritonitis, arthritis, and dermatitis experiments were analyzed by two-way factorial ANOVA. A Mann–Whitney *U* test was used to analyze the body-weight curves. Survival studies were analyzed by the Kaplan–Meier method and logrank statistics. A *p* value < 0.05 was considered statistically significant.

## Results

### Myeloid-specific deletion of Mcl-1 leads to severe neutropenia

To test the effect of myeloid-specific deletion of Mcl-1, we have generated *Mcl1*^ΔMyelo^ mice, which leads to Cre-mediated deletion of *Mcl1* in the myeloid compartment. Control mice included wild type C57BL/6 animals, *Lyz2*^Cre/Cre^ or *Mcl1*^flox/flox^ single-gene mutants, or *Lyz2*^Cre/Cre^*Mcl1*^flox/+^ littermate controls.

**FIGURE 1.**
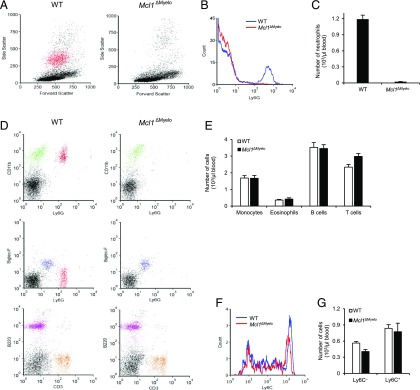
Myeloid-specific deletion of Mcl-1 leads to neutrophil deficiency in peripheral blood. (**A**) Flow cytometric analysis of peripheral blood leukocytes in wild type (WT) and *Mcl1*^ΔMyelo^ mice. Ly6G^+^ cells are indicated with red color. (**B**) Histogram of Ly6G staining of WT and *Mcl1*^ΔMyelo^ peripheral blood leukocytes. (**C**) Quantitative analysis of the number of mature neutrophils (CD11b^+^Ly6G^+^Siglec-F^−^ cells). Flow cytometric profiles (**D**) and quantitative analysis (**E**) of other leukocyte populations (red, neutrophils; green, monocytes; blue, eosinophils; magenta, B cells; orange, T cells). Flow cytometric histograms (**F**) and quantitative analysis (**G**) of monocyte subpopulations. Dot plots and histograms are representative of and quantitative data show mean and SEM from 21 to 28 (A–E) or 13 to 14 (F and G) mice per group from seven (A–E) or five (F and G) independent experiments.

Whereas the peripheral blood of wild type animals contained a clear population of neutrophils (Ly6G^+^ cells with intermediate forward scatter and high side scatter characteristics), this population was missing from *Mcl1*^ΔMyelo^ mice (Fig. 1A, 1B). This was in line with our previously reported experiments with these animals (24, 35). Quantitative analysis (Fig. 1C) revealed that the circulating neutrophil count in the *Mcl1*^ΔMyelo^ mutants was reduced by 98.1% relative to wild type animals (*p* = 8.0 × 10^−23^). No signs of neutropenia were observed in mice carrying mutations only in the *Lyz2* or *Mcl1* gene (Supplemental Fig. 1A). Severe neutropenia was also confirmed by staining peripheral blood neutrophils using the 7/4 or RB6-8C5 (Gr1) markers (Supplemental Fig. 1C, 1D).

### Specificity of the effect of the Mcl1^ΔMyelo^ mutation

We next tested the effect of the *Mcl1*^ΔMyelo^ mutation on other leukocyte lineages. As shown in Fig. 1D and 1E, circulating monocyte (CD11b^+^Ly6G**^−^**Siglec-F^−^; *p* = 0.96), eosinophil (Siglec-F^+^Ly6G^−^; *p* = 0.49), and cell (B220^+^; *p* = 0.86) numbers were normal, and T cell (CD3^+^) numbers were even moderately elevated (*p* = 0.012) in *Mcl1*^ΔMyelo^ mice. Analysis of Ly6C^+^ (“inflammatory”) and Ly6C^−^ (“patrolling”) monocyte subpopulations within the CD11b^+^Ly6G^−^Siglec-F^−^ monocyte gate (Fig. 1F, 1G) indicated normal numbers of Ly6C^+^ monocytes (*p* = 0.73) and a moderate although statistically significant reduction of Ly6C^−^ monocyte counts (*p* = 0.0039). No substantial differences in those lineages were observed when only the *Lyz2* or *Mcl1* genes were mutated (Supplemental Fig. 1A, 1B). No changes in RBC count or blood hemoglobin concentration was observed in *Mcl1*^ΔMyelo^ mice either (data not shown).

### Analysis of tissue leukocytes and in vitro–differentiated macrophages

**FIGURE 2.**
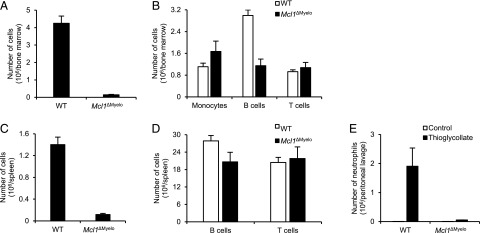
Tissue leukocytes in Mcl1^ΔMyelo^ mice. Tissue neutrophils and other leukocytes were analyzed in wild type (WT) and *Mcl1*^ΔMyelo^ mice from bone marrow, spleen, and peritoneal lavage samples by flow cytometry. Mature neutrophils were identified as CD11b^+^Ly6G^+^ cells. Bar graphs show the absolute number of neutrophils (**A** and **C**) or other leukocytes (**B** and **D**) from the bone marrow (A and B) or the spleen (C and D). (**E**) Quantitative analysis of peritoneal lavage neutrophils after thioglycolate-induced peritonitis or control treatment. Data show mean and SEM from five to six (A–D) or six to eight (E) mice per group from three independent experiments.

We next tested the effect of the *Mcl1*^ΔMyelo^ mutation on tissue leukocyte numbers. As shown in Fig. 2A, the number of Ly6G^+^ neutrophils in the bone marrow was strongly reduced in the *Mcl1*^ΔMyelo^ animals (96% reduction; *p* = 1.1 × 10^−5^). This is also reflected in the strong reduction of the number of cells with neutrophil-like donut-shaped nuclear morphology in cytospin preparations of bone marrow cells (Supplemental Fig. 2A). More detailed analysis of Ly6G expression (Supplemental Fig. 2B) in the bone marrow has revealed that although the Ly6G^high^ population was practically absent in *Mcl1*^ΔMyelo^ mice the Ly6G^med/dim^ populations were not reduced, suggesting that the *Mcl1*^ΔMyelo^ mutation does not eradicate the myeloid progenitor or early neutrophil lineage cell compartment.

In contrast to neutrophils, no reduction of monocytes or T cells could be observed in *Mcl1*^ΔMyelo^ mice (Fig. 2B; *p* = 0.20 and 0.48, respectively). However, the number of bone marrow B cells was clearly reduced (*p* = 4.0 × 10^−4^), despite the fact that circulating B cell numbers were not affected (compare Figs. 1E, 2B). Further analysis of the B cell compartment revealed that this reduction affected all tested B cell populations (proB/preB1, immature, and recirculating B cells; Supplemental Fig. 2C). The fact that even the recirculating B cell counts were reduced despite normal circulating (Fig. 1D, 1E) and splenic (Supplemental Fig. 2D) B cell numbers suggests that the reduced bone marrow B cell counts are likely due to a disturbed bone marrow B cell niche (rather than an intrinsic B cell defect) and that this bone marrow phenotype is well compensated in the periphery. Finally, the analysis of bone marrow macrophages and dendritic cells did not reveal any difference between wild type and *Mcl1*^ΔMyelo^ mice either (Supplemental Fig. 2E).

We have also tested various splenic leukocyte populations. As shown in Fig. 2C, splenic neutrophil numbers were strongly reduced in *Mcl1*^ΔMyelo^ animals (93% reduction; *p* = 1.5 × 10^−6^). However, as shown in Fig. 2D, the number of splenic T or B cells was not affected (*p* = 0.77 and 0.092, respectively). Further analysis of splenic B cells (Supplemental Fig. 2D) also failed to reveal a defect in any of the splenic B cell populations tested. Additional studies on splenic macrophages and dendritic cells failed to reveal any reduction in their numbers in *Mcl1*^ΔMyelo^ mice (Supplemental Fig. 2F). However, the number of splenic macrophages was significantly increased in *Mcl1*^ΔMyelo^ animals (Supplemental Fig. 2F), which correlated with the size of the spleen in those mice (i.e., the difference disappeared after normalization for the weight of the spleen). Therefore, we believe that the increased macrophage number is related to splenomegaly in those mice (see below), reflecting the fact that macrophages represent one of the predominant cell types in this organ.

The number of tissue neutrophils under inflammatory conditions was assessed in thioglycolate-induced peritonitis. As shown in Fig. 2E, thioglycolate injection triggered a robust neutrophil infiltration in wild type animals, whereas no such infiltration could be observed in *Mcl1*^ΔMyelo^ mice (97% reduction; *p* = 1.3 × 10^−4^). Therefore, the severe neutrophil deficiency in *Mcl1*^ΔMyelo^ mice is also evident under inflammatory conditions.

We have also tested the in vitro differentiation of macrophages from *Mcl1*^ΔMyelo^ bone marrow cells. We did not observe any difference between the number of bone marrow–derived macrophages generated from wild type or *Mcl1*^ΔMyelo^ bone marrow cells (Supplemental Fig. 2G), and the morphology and F4/80 expression profile was also similar between those genotypes (data not shown). In contrast, PCR analysis of genomic DNA confirmed effective deletion of the *Mcl1*^flox^ allele in bone marrow–derived macrophage cultures, whereas only a marginal deletion (likely because of the presence of tissue macrophages or osteoclasts) was seen in tail biopsy samples (Supplemental Fig. 2H; see further explanation in the figure legend). Those results indicate that *Mcl1* deletion does not affect the proliferation, differentiation, or overall morphology of macrophages.

### Survival of Mcl1^ΔMyelo^ mice

Although it is generally believed that severe neutropenia is inconsistent with life, this has never been tested in mice, in part because of the limitations of currently existing neutropenic mouse models (16–20). Therefore, we tested the survival of the *Mcl1*^ΔMyelo^ mice during a prolonged period of time.

**FIGURE 3.**
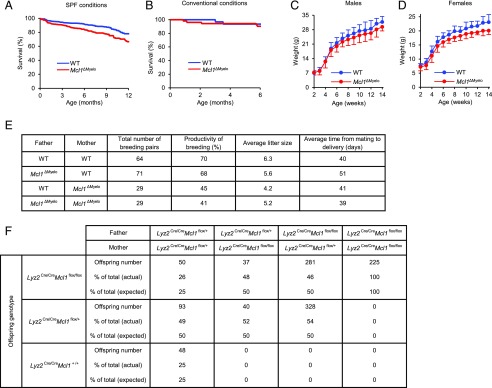
Survival and fertility of Mcl1^ΔMyelo^ mice. (**A** and **B**) Survival of wild type (WT) and *Mcl1*^ΔMyelo^ mice under specific pathogen-free (SPF) (A) or conventional (B) conditions. (**C** and **D**) The body weight of WT and *Mcl1*^ΔMyelo^ male (C) and female (D) mice. (**E**) Breeding behavior of WT and *Mcl1*^ΔMyelo^ mice. Breeding was considered productive when pups were born from a given mating. (**F**) Genotype distribution of offspring from different breeding strategies. Survival curves show data of 611–977 (A) or 31–52 (B) mice per group, whereas body weight analysis shows mean and SD from 7 to 28 (C) or 9 to 26 (D) mice per group. Data from 193 breeding pairs and 1520 pups were used for the analysis of breeding behavior and offspring genotype.

Surprisingly, and in contrast to our previous assumptions, the survival of *Mcl1*^ΔMyelo^ mice under specific pathogen-free conditions was not dramatically different from that of wild type animals (Fig. 3A). Although there was a moderate reduction of the survival of *Mcl1*^ΔMyelo^ mice compared with wild type animals (84% versus 92% at 6 mo and 66% versus 78% at 12 mo of age, respectively) and this was statistically highly significant (*p* < 0.00001) due to the very large number of mice tested (>600 per genotype), this difference was not at all dramatic, especially at the early age range when most animal experiments are performed.

The effect of the *Mcl1*^ΔMyelo^ mutation under more real-world conditions was tested on a smaller cohort of mice in a conventional animal facility (Fig. 3B). Importantly, the survival of *Mcl1*^ΔMyelo^ animals was again only slightly below that of the wild type mice (88 and 93% at 6 mo of age, respectively; *p* = 0.032), indicating that the survival of *Mcl1*^ΔMyelo^ mice is not dramatically affected even under conventional conditions.

We did not see any substantial difference between the general appearance or behavior of wild type and *Mcl1*^ΔMyelo^ mice (data not shown). Body weight measurements revealed a slight reduction in *Mcl1*^ΔMyelo^ mice (Fig. 3C, 3D; *p* = 0.22 and 2.0 × 10^−6^ for males and females, respectively). The only consistent difference found during dissection was splenomegaly in *Mcl1*^ΔMyelo^ animals, which appeared to become more severe in older animals (data not shown).

### Mcl1^ΔMyelo^ mice breed in homozygous form

We also tested the breeding behavior of *Mcl1*^ΔMyelo^ animals in our specific pathogen-free facility. As shown in Fig. 3E, new pups were born from all mating strategies (even when both parents were of *Mcl1*^ΔMyelo^ genotype) although the overall productivity of the breeding was reduced when *Mcl1*^ΔMyelo^ females were used. Most importantly, breeding *Mcl1*^ΔMyelo^ in homozygous form still yielded a comparable number of offspring as wild type breeding pairs, and the moderate reduction was not substantially more severe than what is usually observed during breeding of other genetically manipulated mice. We were also able to breed a smaller cohort of *Mcl1*^ΔMyelo^ mice in homozygous form in our conventional facility (data not shown). Analysis of the genotype of the offspring was also very close to the expected Mendelian ratios in all cases (Fig. 3F), indicating normal embryonic and early postnatal survival of *Mcl1*^ΔMyelo^ mice.

Taken together, our results indicate that the *Mcl1*^ΔMyelo^ mice are viable and fertile even in homozygous mutant form, both under specific pathogen-free and conventional conditions. In addition to the surprising finding of practically normal survival in the almost complete absence of circulating neutrophils (Figs 1, 2), these results also indicate that the *Mcl1*^ΔMyelo^ mouse strain may be relatively easy to maintain and, therefore, may be a technically very useful model for the in vivo analysis of neutrophil function. This is particularly true given that no individual offspring genotyping is needed upon homozygous breeding and that most mouse experiments are performed on younger animals, in which the survival effect of the *Mcl1*^ΔMyelo^ mutation is marginal.

### Defective autoantibody-mediated inflammation in Mcl1^ΔMyelo^ mice

The functional relevance of neutropenia in *Mcl1*^ΔMyelo^ mice was tested in two autoantibody-induced, supposedly neutrophil-dependent in vivo inflammation models.

**FIGURE 4.**
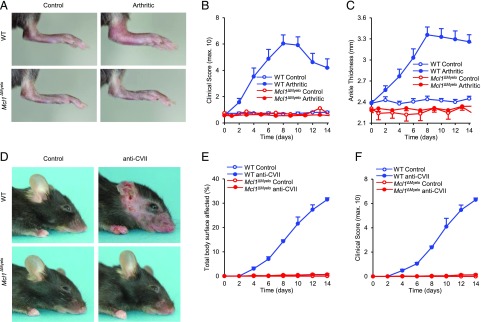
Autoantibody-induced arthritis and skin-blistering disease in Mcl1^ΔMyelo^ mice. (**A**–**C**) Wild type (WT) or *Mcl1*^ΔMyelo^ mice were injected with control (B×N) or arthritic (K/B×N) serum on day 0. Arthritis development was followed by photographing on day 7 (A), clinical scoring of the hind limbs (B) and ankle thickness measurement (C). (**D**–**F**) Skin-blistering disease was triggered in wild type (WT) or *Mcl1*^ΔMyelo^ mice by systemic injection of control IgG or CVII-specific (anti-CVII) Abs. Skin disease was followed by photographing on day 14 (D) and clinical assessment of the total body surface affected (E) and the overall disease severity (F). Images are representative of and quantitative data show mean and SEM from five to nine control and 9 to 15 arthritic serum–treated individual mice per group from three independent experiments (A–C), or from three to four control and three to four anti-CVII–treated mice per genotype from two independent experiments (D–F).

Mice were first subjected to K/B×N serum-transfer arthritis, an autoantibody-induced in vivo arthritis model (36, 37) previously suggested to be mediated by neutrophils (38, 39). As shown in Fig. 4A, K/B×N serum injection triggered robust arthritis in wild type mice, whereas *Mcl1*^ΔMyelo^ mutants appeared to be completely protected. Kinetic analysis of clinical score (Fig. 4B; *p* = 4.2 × 10^−5^) and ankle thickness (Fig. 4C; *p* = 0.0059) has confirmed those findings. The protection of *Mcl1*^ΔMyelo^ mice was not due to deletion of LysM by the *Lyz2*^Cre/Cre^ knock-in mutation because K/B×N serum-transfer arthritis developed normally in *Lyz2*^Cre/Cre^ mice (Supplemental Fig. 3).

Neutrophils have been proposed to be critical for the development of anti-CVII Ab–induced dermatitis, a mouse model of the rare human-blistering skin disease epidermolysis bullosa acquisita (33, 40, 41). Anti-CVII Abs triggered severe skin inflammation in wild type mice, whereas no signs of the disease could be observed in *Mcl1*^ΔMyelo^ animals (Fig. 4D). Kinetic analysis revealed that *Mcl1*^ΔMyelo^ mice were completely protected from skin inflammation, both in terms of the affected body surface (Fig. 4E; *p* = 3.8 × 10^−11^) and of a more elaborate clinical scoring system (Fig. 4F; *p* = 3.9 × 10^−10^).

Taken together, our results indicate that *Mcl1*^ΔMyelo^ mice are completely protected from two separate, neutrophil-mediated autoantibody-induced inflammation models.

### Increased susceptibility to bacterial and fungal infection

Although *Mcl1*^ΔMyelo^ mice resisted the microbial burden of their commensal flora (Fig. 3), we wanted to test their susceptibility to experimentally induced infections. Therefore, we subjected our mice to systemic *S. aureus* or *C. albicans* infection.

**FIGURE 5.**
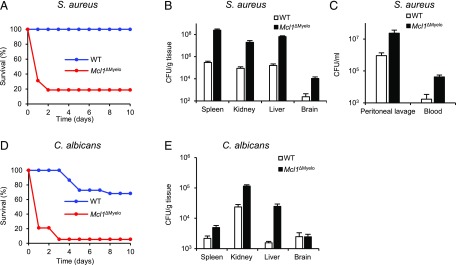
Mcl1^ΔMyelo^ mice are highly susceptible to bacterial and fungal infections. (**A**–**C**) Survival curves (A) or analysis of the bacterial burden from the indicated tissues (B and C) of wild type (WT) and *Mcl1*^ΔMyelo^ mice following i.p. injection with 2 × 10^7^ (A) or 10^7^ (B and C) *S. aureus* bacteria. (**D** and **E**) Survival curves (D) or analysis of the fungal burden from the indicated tissues (E) of WT and *Mcl1*^ΔMyelo^ mice following i.v. injection with 10^5^ *C. albicans*. Survival curves show the data of 16 (A) or 19–22 (D) mice per group from three independent experiments. Bar graphs show mean and SEM from 9 to 10 (B and C) or 10 to 11 (E) mice per group from three (B and C) or four (E) independent experiments.

Neutrophils are the major players in the host defense against infections by *S. aureus*, a Gram-positive pathogen able to cause skin and respiratory tract infection, abscess formation, and bacteremia/sepsis (42, 43). As shown in Fig. 5A, whereas wild type animals survived i.p. infection with 2 × 10^7^ *S. aureus*, more than 80% of *Mcl1*^ΔMyelo^ mice succumbed to the same infectious challenge (*p* = 1.0 × 10^−5^). Analysis of the bacterial burden 12 h after the infection with 1 × 10^7^ bacteria revealed a more than 100-fold increase of bacterial colony counts in the spleen (*p* = 0.0015), kidneys (*p* = 0.023), and liver (*p* = 9.0 × 10^−5^) and significant increases in the brain (*p* = 0.028) and in the blood (*p* = 0.0038) but not in the peritoneum (*p* = 0.098) of *Mcl1*^ΔMyelo^ mice (Fig. 5B, 5C).

Neutrophils are among the critical immune cells protecting the host from infection by *C. albicans*, a fungal pathogen able to cause superficial or systemic infections and one of the most prevalent causes of hospital-acquired infections (44). As shown in Fig. 5D, i.v. infection with 10^5^ *C. albicans* caused lethality in 27% of wild type animals, whereas the same infection caused rapid lethality in 95% of *Mcl1*^ΔMyelo^ mice (*p* < 0.00001). Analysis of the fungal burden at 12 h revealed a more than 10-fold increase in fungal counts in the liver (*p* = 1.0 × 10^−4^) of *Mcl1*^ΔMyelo^ mice, with moderate increase also in the spleen (*p* = 0.0096) and in the kidneys (*p* = 5.2 × 10^−7^) but not in the brain (*p* = 0.97) of the animals (Fig. 5E).

Taken together, *Mcl1*^ΔMyelo^ mice are highly susceptible to infectious challenge by bacterial or fungal pathogens, such as *S. aureus* or *C. albicans*, likely because of defective neutrophil-mediated elimination of the pathogens.

### Analysis of Mcl1^ΔMyelo^ bone marrow chimeras

**FIGURE 6.**
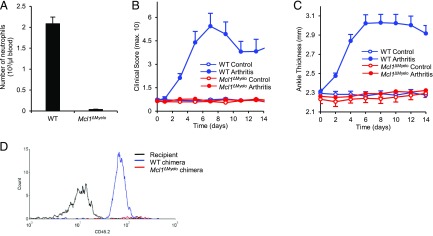
Neutrophil deficiency and autoantibody-induced arthritis in Mcl1^ΔMyelo^ bone marrow chimeras. (**A**) Number of circulating neutrophils (CD11b^+^Ly6G^+^Siglec-F^–^ cells) of wild type (WT) or *Mcl1*^ΔMyelo^ bone marrow chimeras by flow cytometry. (**B** and **C**) Analysis of the clinical score (B) and ankle thickness (C) of WT and *Mcl1*^ΔMyelo^ bone marrow chimeras injected with control (B×N) or arthritic (K/B×N) serum on day 0. (**D**) Representative flow cytometric analysis of donor marker (CD45.2) expression in circulating neutrophils (Ly6G^+^ gate) from intact (nonchimeric) mice of the CD45.1-expressing recipient strain as well as from wild type (WT) or *Mcl1*^ΔMyelo^ bone marrow chimeras. A representative histogram from a large number of experiments is shown. Quantitative data show mean and SEM from 17 chimeras (A) or from eight control and nine arthritic serum–treated chimeras per group from two (A) or three (B and C) independent experiments.

It is often difficult to obtain larger homogeneous cohorts of mice for in vivo experiments from small breeding colonies. When studying neutrophil function, this problem may be overcome by transplanting bone marrow cells to larger cohorts of recipient mice. To test that possibility, we transplanted wild type or *Mcl1*^ΔMyelo^ bone marrow cells into lethally irradiated wild type recipients carrying the CD45.1 allele. As shown in Fig. 6D, circulating neutrophils from such chimeras consisted practically exclusively of CD45.2-expressing cells (i.e., donor cells carrying the CD45.2 allele from the C57BL/6 genetic background), indicating successful replacement of the recipients’ hematopoietic compartment by donor-derived cells. As shown in Fig. 6A, circulating neutrophil numbers of *Mcl1*^ΔMyelo^ bone marrow chimeras was strongly reduced compared with parallel-generated wild type chimeras (98.3% reduction; *p* = 1.8 × 10^−14^). *Mcl1*^ΔMyelo^ bone marrow chimeras were also completely protected from K/B×N serum-transfer arthritis, both in terms of clinical score (*p* = 2.1 × 10^−5^; Fig. 6B) and ankle thickness changes (*p* = 2.2 × 10^−6^; Fig. 6C). Therefore, bone marrow transplantation can be used to generate larger cohorts of mice with neutropenia caused by the *Mcl1*^ΔMyelo^ mutation.

### Neutrophil-specific deletion of Mcl-1 leads to neutropenia with severe survival defects

The above experiments were performed using *Mcl1*^ΔMyelo^ mice in which Mcl-1 was deleted from the entire myeloid compartment. To test the effect of Mcl-1 deletion in a more neutrophil-specific manner, we have crossed the *Mcl1*^flox/flox^ mice to mice carrying the MRP8-Cre transgene, which drives Cre expression specifically in the neutrophil compartment (45).

**FIGURE 7.**
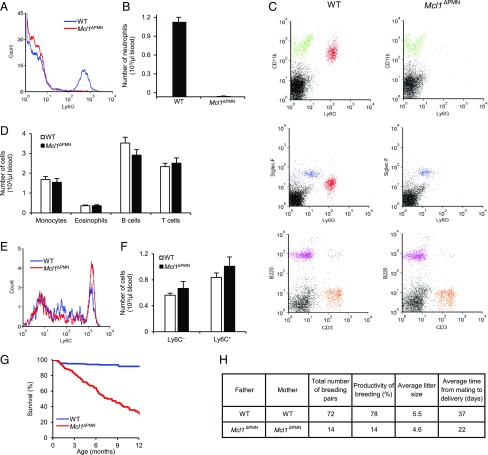
Neutrophil-specific deletion of Mcl-1 leads to neutrophil deficiency with survival and breeding defects. (**A**) Flow cytometric histograms of Ly6G staining of wild type (WT) and *Mcl1*^ΔPMN^ mouse peripheral blood leukocytes. (**B**) Quantitative analysis of the number of mature neutrophils (CD11b^+^Ly6G^+^Siglec-F cells) in WT and *Mcl1*^ΔPMN^ mice. Flow cytometric profiles (**C**) and quantitative analysis (**D**) of other leukocyte populations (red, neutrophils; green, monocytes; blue, eosinophils; magenta, B cells; orange, T cells). Flow cytometric histograms (**E**) and quantitative analysis (**F**) of monocyte subpopulations. (**G**) Survival of WT and *Mcl1*^ΔPMN^ mice under specific pathogen-free conditions. (**H**) Breeding behavior of WT and *Mcl1*^ΔPMN^ mice. Flow cytometry dot plots and histograms are representative of and quantitative data show mean and SEM from, 10–22 (A–D) or 8–14 (E and F) mice per group from four (A–D) or three (E and F) independent experiments. Survival curves (G) show the data of 138–469 mice per genotype. Eighty-six breeding pairs were used for the analysis of breeding behavior (H).

*Mcl1*^ΔPMN^ mice showed dramatic (99.1%, *p* = 9.8 × 10^−12^) reduction of circulating neutrophil counts (Fig. 7A, 7B) that was even more severe than the reduction seen in *Mcl1*^ΔMyelo^ animals (98.1%; see Fig. 1). The *Mcl1*^ΔPMN^ mutation did not affect circulating monocyte (*p* = 0.60), eosinophil (*p* = 0.99), B cell (*p* = 0.21), or T cell (*p* = 0.58) numbers (Fig. 7C, 7D) or the distribution of monocytes into Ly6C^+^ (inflammatory) or Ly6C^−^ (patrolling) monocytes (*p* = 0.24 and 0.26, respectively; Fig. 7E, 7F). Therefore, similar to the *Mcl1*^ΔMyelo^ mutation, the *Mcl1*^ΔPMN^ mutation also leads to severe and selective neutropenia.

Analysis of the survival of *Mcl1*^ΔPMN^ mice (Fig. 7G) revealed a steady and substantial loss of *Mcl1*^ΔPMN^ animals, leading to 58% survival at 6 mo and only 30% survival at 12 mo of age (*p* < 0.000001). *Mcl1*^ΔPMN^ mice were also clearly distinguishable from their wild type littermates and often showed a severe wasting phenotype (data not shown). *Mcl1*^ΔPMN^ mice also showed a very poor breeding productivity (Fig. 7H). Taken together, our data suggest that the limited survival and breeding capacity makes *Mcl1*^ΔPMN^ mice rather difficult to maintain. This is further complicated by the fact that such poor breeders need to be maintained in heterozygous form; therefore, all offspring need to be individually genotyped, and only a fraction of the pups (25% in the most sensible *MRP8*-Cre*Mcl1*^flox/+^ × *Mcl1*^flox/flox^ breeding strategy) are expected to be of the desired *Mcl1*^ΔPMN^ genotype.

Bone marrow transplantation experiments revealed that *Mcl1*^ΔPMN^ bone marrow chimeras also showed a severe wasting phenotype and succumbed to death 3–8 wk after transplantation (data not shown). Although initial results indicated complete protection of *Mcl1*^ΔPMN^ mice from K/B×N serum-transfer arthritis, the limited availability and fragile health status of those mice did not allow us to complete a sufficient number of those experiments (data not shown). The same issue also prevented us from performing more detailed analysis of the tissue leukocyte populations in *Mcl1*^ΔPMN^ mice. Nevertheless, it is interesting to note that in contrast to *Mcl1*^ΔMyelo^ mice the few *Mcl1*^ΔPMN^ animals we were able to dissect did not show an overt splenomegaly phenotype (data not shown).

Taken together, our results indicate that although the *Mcl1*^ΔPMN^ mutation leads to severe and specific neutropenia, the poor and fragile health status, limited survival and fertility, and nonhomozygous nature of those animals makes them hardly suitable for larger-scale in vivo experiments.

### Partial neutropenia in G-CSF receptor–deficient mice

We have also tested G-CSF receptor–deficient (*Csf3r*^−/−^) mice (20) as a reference neutropenic mouse strain. *Csf3r*^−/−^ mice showed only partial neutropenia (*p* = 1.1 × 10^−4^; Supplemental Fig. 4A, 4B), which was not nearly as severe and consistent as in *Mcl1*^ΔMyelo^ or *Mcl1*^ΔPMN^ animals (Figs. 1, 7). Similar to the *Mcl1*^ΔMyelo^ and *Mcl1*^ΔPMN^ mutations, the *Csf3r*^−/−^ mutation did not affect other circulating leukocyte populations either (Supplemental Fig. 4C, 4D). Although the survival of *Csf3r*^−/−^ mice was not substantially reduced, we also had difficulties breeding *Csf3r*^−/−^ mice in homozygous form (data not shown). Those results indicate severe limitations of the *Csf3r*^−/−^ mutation as a neutropenia model.

## Discussion

Our results indicate that *Mcl1*^ΔMyelo^ mice lacking Mcl-1 in the myeloid lineage are severely neutropenic but survive and breed in homozygous form. Those mice may, therefore, be highly useful in analyzing the role of neutrophils in in vivo processes in health and disease. Our results also indicate that mice are able to survive almost normally when the circulating neutrophil numbers are reduced to <2% of their normal values, necessitating the re-evaluation of the role of neutrophils in rodent survival.

Currently available tools for reducing neutrophil numbers have substantial limitations. Although Ab-mediated depletion (e.g., by the RB6-8C5 or NIMP-R14 anti-Gr1 or the 1A8 anti–Ly6G Abs) has clear benefits, such as easy availability and suitability to be used on transgenic strains without breeding delay, it suffers from limited specificity (especially when using anti-Gr1 Abs), very high reagent costs, and the temporary nature of the depletion. Prior reports of neutropenic mice (16–21) also revealed phenotypes that strongly limit their use as in vivo neutropenia models. Besides severe neutropenia, *Gfi1*-deficient mice also show various defects in the T and B cell compartment and have a median survival time of ∼8–10 wk (16, 17), in line with the severe neutropenia and lymphocyte defects caused by dominant negative *GFI1* mutations in human patients (46). The so-called “Genista” mice carrying a chemically induced *Gfi1* mutation show incomplete neutrophil deficiency and are only partially protected in a neutrophil-dependent in vivo inflammation model (18). Mice lacking G-CSF (19) or the G-CSF receptor (20) are only moderately neutropenic (see also Supplemental Fig. 4), and the latter strain also shows breeding defects (data not shown). Deficiency of the Foxo3A transcription factor causes accelerated neutrophil apoptosis at the site of inflammation but does not affect circulating neutrophil numbers (21). In contrast to those genetic and pharmacological models, the *Mcl1*^ΔMyelo^ mice show consistent, severe, and fairly specific neutropenia and survive and breed in homozygous form, making them quite useful as an in vivo neutropenia model.

The specificity of reduced neutrophil numbers in *Mcl1*^ΔMyelo^ mice is due to two factors: the deletion of the antiapoptotic Mcl-1 protein in the entire myeloid lineage (including macrophages) and the specific requirement for Mcl-1 for the survival of neutrophils but not of the cells of the monocyte/macrophage lineage (24, 47). This is also indicated by the normal number and overall appearance of macrophages differentiated from *Mcl1*^ΔMyelo^ bone marrow cells, despite effective deletion of the *Mcl1*^flox^ allele (Supplemental Fig. 2G, 2H). We have also tested *Mcl1*^ΔPMN^ mice in which *Mcl1* deletion is achieved by the *MRP8*-Cre transgene, which is more specific for neutrophils than the *Lyz2*^Cre^ knock-in mutant (45). Although the *Mcl1*^ΔPMN^ mutations also strongly reduced circulating neutrophil counts and appeared to be specific over several other leukocyte lineages (Fig. 7), the limited survival and poor breeding of those mice make them very difficult to use as an in vivo neutropenia model. Although it is at present unclear why the *Mcl1*^ΔMyelo^ and *Mcl1*^ΔPMN^ mice have different survival and breeding characteristics, one of the possible explanations is that the remaining ∼2% of neutrophils in *Mcl1*^ΔMyelo^ mice is sufficient to control the commensal flora, whereas the ∼1% of remaining neutrophils in the *Mcl1*^ΔPMN^ mutants is below the threshold of neutrophil levels required for normal survival. It would theoretically also be possible that the survival of the *Mcl1*^ΔMyelo^ mice is due to some genetic drift in our mouse colony, although our heterozygous breeding strategy argues against that possibility. Understanding the exact reason for the different survival of *Mcl1*^ΔMyelo^ and *Mcl1*^ΔPMN^ mice would require substantial additional experiments, including detailed apoptosis and in vitro progenitor differentiation/proliferation assays.

Although the *Mcl1*^ΔMyelo^ mutation causes severe neutropenia both in the peripheral blood and in various tissues (Figs. 1, 2), it is at present not entirely clear at which stage the mutation interferes with neutrophil development and/or survival. The fact that the number of Ly6G^med/dim^ cells in the bone marrow is not reduced (Supplemental Fig. 2B) suggests that the *Mcl1*^ΔMyelo^ mutation affects cells in the latest stage of neutrophil development. This is also supported by the fact that HoxB8-transduced myeloid progenitors were unable to engraft the bone marrow of *Mcl1*^ΔMyelo^ mice (A.O. and A.M., unpublished observations), suggesting that the myeloid progenitor niche is preoccupied by endogenous cells in those animals.

Although the *Mcl1*^ΔMyelo^ mutation proved to be fairly specific for neutrophils, we have consistently observed reduced B lineage cell numbers in the bone marrow of *Mcl1*^ΔMyelo^ mice (Fig. 2B). The relevance of this finding is at present unclear, especially given the normal circulating (Fig. 1E) and splenic (Fig. 2D) B cell counts. More detailed analysis of the B cell compartment (Supplemental Fig. 2C, 2D) has revealed that even recirculating B cell numbers are reduced in the bone marrow of *Mcl1*^ΔMyelo^ animals, suggesting disturbance of the bone marrow B cell niche. Alternatively, this observation may be due to the expression of LysM in the early B cell lineage as indicated by the ImmGen database (www.immgen.org). It should also be noted that more splenic macrophages (Supplemental Fig. 2F) were observed in *Mcl1*^ΔMyelo^ mice, which likely reflects splenomegaly in those animals.

To our knowledge, this is the first detailed characterization and validation of the *Mcl1*^ΔMyelo^ mice as a suitable experimental neutropenia model. In particular, our study provides the most detailed lineage analysis of those animals, reports large-scale assessment of their survival and fertility, and validates the mutant mice on known neutrophil-dependent in vivo inflammation and infection models. To our knowledge, we also provide the first detailed analysis of *Mcl1*^ΔPMN^ mice and a side-by-side comparison of the *Mcl1*^ΔMyelo^, *Mcl1*^ΔPMN^, and *Csf3r*^−/−^ mutants. It should, nevertheless, be noted that we have already used the *Mcl1*^ΔMyelo^ model in the recent past to test the role of neutrophils in various disease models, such as graft-versus-host disease (48), contact hypersensitivity (35), gout (49), and experimental lupus (50). All those reports and further ongoing studies have confirmed the usefulness of this model for the in vivo analysis of neutrophil function.

Taken together, our results indicate that the unique combination of severe and fairly specific neutropenia, mostly normal survival, and capability for breeding in homozygous form make the *Mcl1*^ΔMyelo^ mutation highly suitable for the analysis of the role of neutrophils in in vivo models of normal and pathological processes in experimental mice. Our results also indicate that rodents are able to survive and breed when their circulating neutrophil counts are dramatically reduced.

## Supplementary Material

Data Supplement
