# Supplementary material for: Myeloid-Specific Deletion of Mcl-1 Yields Severely Neutropenic Mice That Survive and Breed in Homozygous Form
Source: J Immunol. 2018 Nov 21;201(12):3793–803. doi: 10.4049/jimmunol.1701803 (PMC6287103; doi:10.4049/jimmunol.1701803)
Supplement: Data Supplement [file JI_1701803.zip › JI_1701803_Supplemental_Figures_1.pdf]

# Supplementary material

## Myeloid-specific deletion of Mcl-1 yields severely neutropenic mice which survive and breed in homozygous form

Janka Zsófia Csepregi, Anita Orosz, Erik Zajta, Orsolya Kása, Tamás Németh, Edina Simon, Szabina Fodor, Katalin Csonka, Balázs L. Barátki, Dorottya Kövesdi, Youwen He, Attila Gácser and Attila Mócsai

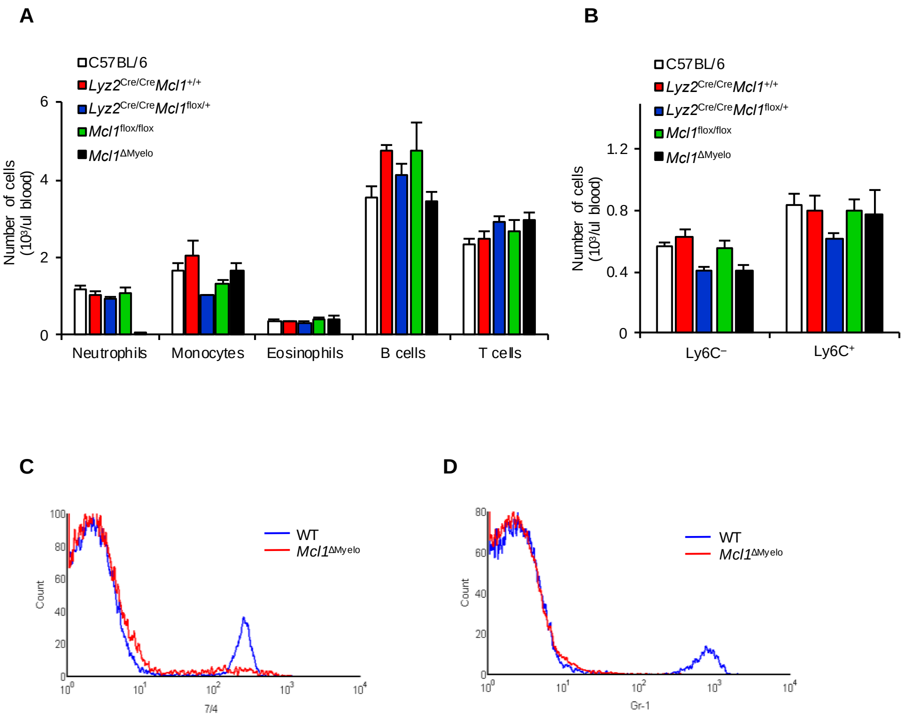


**Figure S1**

*Analysis of peripheral leukocytes in various mutant mouse strains*

Circulating neutrophils and other leukocytes were tested in C57BL/6, *Lyz2*$^{\text{Cre/Cre}}$*Mcl1*$^{+/+}$, *Lyz2*$^{\text{Cre/Cre}}$*Mcl1*$^{\text{flox/+}}$, *Mcl1*$^{\text{flox/flox}}$ and *Mcl1*$^{\Delta\text{Myelo}}$ mice by flow cytometry. (A) Quantitative analysis of the number of the indicated leukocyte subsets. (B) Quantitative analysis of the number of Ly6G$^+$ and Ly6G$^-$ monocyte subpopulations. (C) Flow cytometric analysis of circulating neutrophil counts in wild type (WT) or *Mcl1*$^{\Delta\text{Myelo}}$ mice stained with 7/4 (C) or RB6-8C5 (Gr-1; D) antibodies. Quantitative data show mean and SEM from, and flow cytometry histograms are representative of, 8-22 (A), 8-13 (B) or 2 (C, D) mice per group from 3 (A,B) or 2 (C, D) independent experiments.

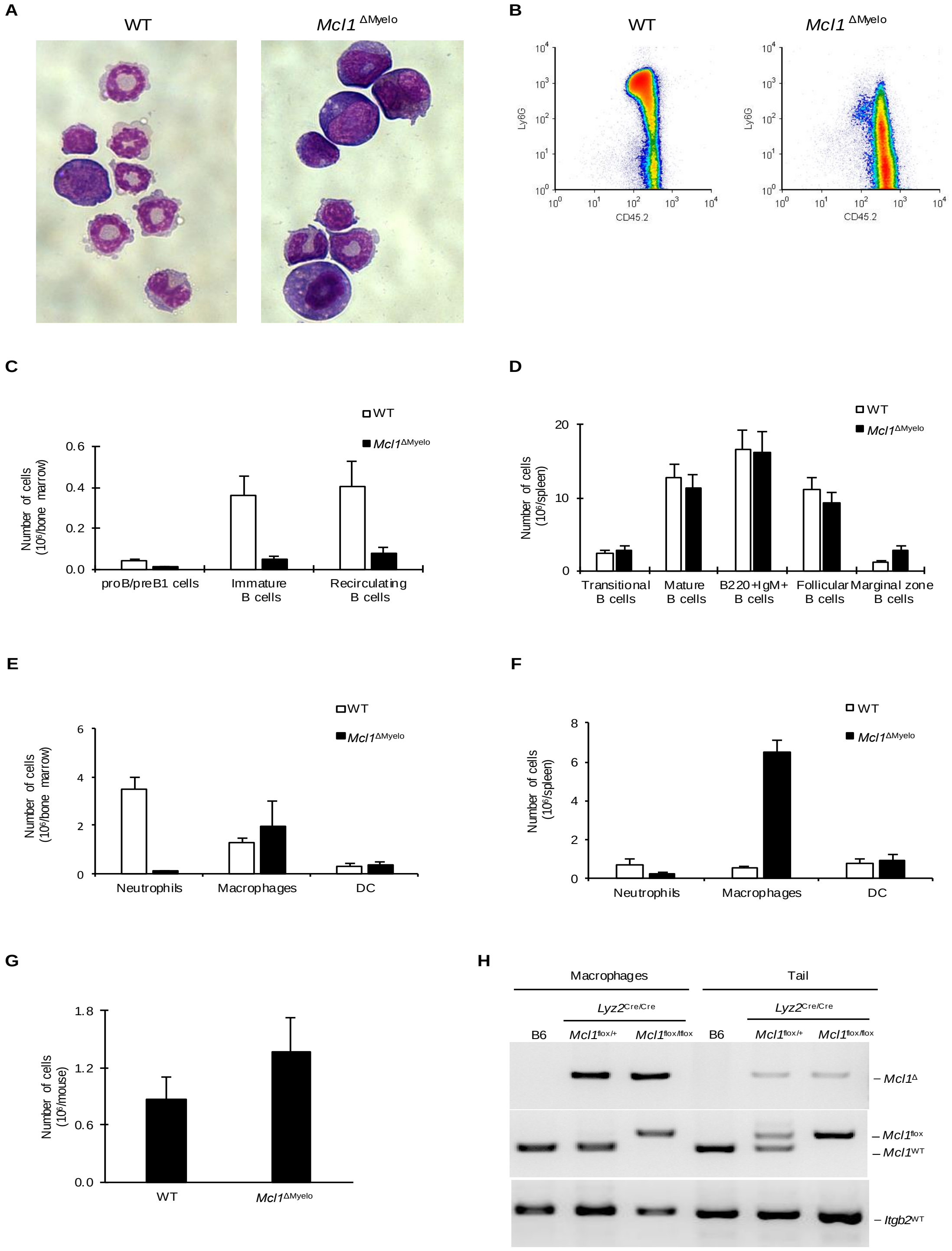


**Figure S2**

*Analysis of tissue leukocytes and in vitro differentiated macrophages*

A-F, bone marrow and splenic cells of wild type (WT) or *Mcl1*$^{\Delta\text{Myelo}}$ mice were analyzed. (A) Representative cell morphologies of WT and *Mcl1*$^{\Delta\text{Myelo}}$ mouse bone marrow cells analyzed by the cytospin approach. (B) Density plots of WT and *Mcl1*$^{\Delta\text{Myelo}}$ mouse bone marrow cells, characterized by Ly6G and CD45 (CD45.2) staining and flow cytometry. (C) B cell subpopulations of the bone marrow from WT and *Mcl1*$^{\Delta\text{Myelo}}$ mice, characterized by c-kit$^{+}$B220$^{\text{med}}$ (proB/preB1 cells), IgM$^{+}$B220$^{\text{med}}$ (immature B cells) and IgM$^{+}$B220$^{+}$ (recirculating B cells) staining by flow

cytometry. (D) B cell subpopulations in the spleen of WT and *Mcl1*$^{\Delta Myelo}$ mice, characterized by IgM$^{+}$IgD$^{+}$ (mature B cells), IgM$^{+}$IgD$^{med}$ (transitional B cells), CD21$^{+}$CD23$^{+}$ (follicular B cells) and CD21$^{+}$CD23$^{med}$ (marginal zone B cells) staining by flow cytometry. (E) Macrophages and dendritic cells (DC) from the bone marrow and spleen (F) of WT and *Mcl1*$^{\Delta Myelo}$ mice. Macrophages were identified as CD45$^{+}$F4/80$^{+}$ cells and DC were identified as CD45$^{+}$iA/B$^{+}$CD11c$^{+}$ cells by flow cytometry. G-H, analysis of bone marrow-derived macrophages from C57BL/6 (B6), *Lyz2*$^{Cre/Cre}$*Mcl1*$^{flox/+}$ and *Mcl1*$^{\Delta Myelo}$ (*Lyz2*$^{Cre/Cre}$*Mcl1*$^{flox/flox}$) mice. (G) Macrophage cell counts were tested by flow cytometry. Mature macrophages were identified as F4/80$^{+}$ cells. (H) PCR-based analysis of Cre mediated deletion of the *Mcl1* gene of in vitro cultured macrophages and tail samples. *Mcl1*$^{\Delta}$ indicates Cre-mediated deletion of the *Mcl1*$^{flox}$ allele. The *Mcl1*$^{flox}$ and *Mcl1*$^{WT}$ PCR protocol used the same primer set with different product lengths. The *Itgb2*$^{WT}$ PCR was used as an irrelevant control. Because of the saturating nature of PCR reactions, the deletion of the *Mcl1*$^{flox}$ allele is best seen in the *Lyz2*$^{Cre/Cre}$*Mcl1*$^{flox/+}$ samples. Here, the practically complete lack of the *Mcl1*$^{flox}$ PCR product indicates strong deletion of the floxed allele in macrophages, as compared to the normal presence of the *Mcl1*$^{flox}$ allele in tail biopsies. The obvious presence of the *Mcl1*$^{flox}$ PCR product in the *Mcl1*$^{\Delta Myelo}$ macrophage samples likely reflects efficient PCR-based amplification of a low amount of the *Mcl1*$^{flox}$ allele still present in these samples in the absence of competing *Mcl1*$^{WT}$ sequences, rather than the lack of deletion of the *Mcl1*$^{flox}$ allele, as indicated by the strong *Mcl1*$^{\Delta}$ product present in those samples. Images are representatives of, and quantitative data show mean and SEM from, 1 (A) or 2-3 mice (B-F) per group from 2-3 independent experiments.

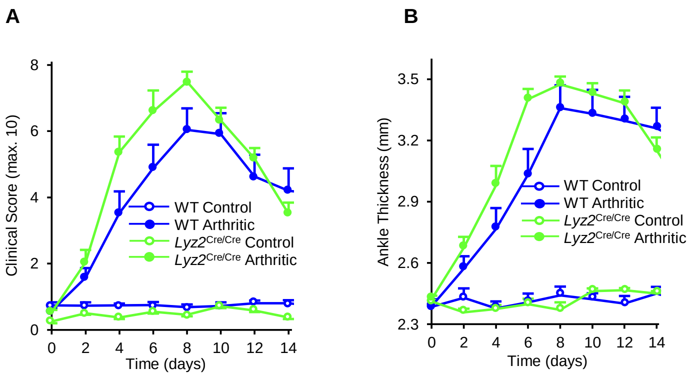


**Figure S3**

*Autoantibody-induced arthritis in Lyz2*$^{Cre/Cre}$ *mice*

Wild type (WT) or *Lyz2*$^{c/c}$ mice were injected with control (B×N) or arthritic (K/B×N) serum on Day 0. Arthritis development was followed by clinical scoring of the hind limbs (A) and ankle thickness measurement (B). Data show mean and SEM from 3-9 control and 5-15 arthritic serum-treated individual mice per group from 3 independent experiments.

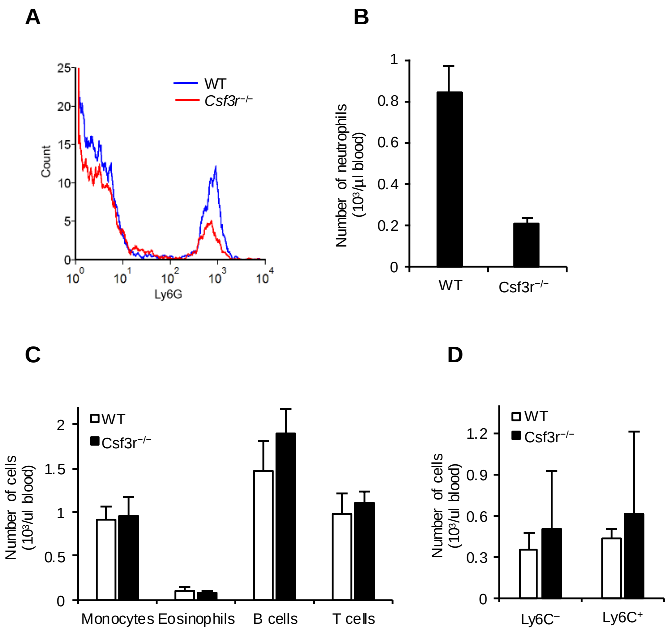


**Figure S4**

*Moderate neutrophil deficiency in Csf3r*$^{-/-}$ *mice*

(A) Flow cytometric histograms of Ly6G staining of peripheral blood leukocytes of wild type (WT) and *Csf3r*$^{-/-}$ mice. (B) Quantitative analysis of the number of mature neutrophils (CD11b$^{+}$Ly6G$^{+}$Siglec-F$^{-}$). (C, D) Quantitative analysis of the number of other leukocyte populations (C) and monocyte subpopulations (D). Flow cytometry histograms are representative of, and quantitative data show mean and SEM from, 8 (A, B, C) or 4 (D) mice per group from 3 (A, B, C) or 2 (D) independent experiments.
